# Retraction: Foliar application of potassium and moringa leaf extract improves growth, physiology and productivity of kabuli chickpea grown under varying sowing regimes

**DOI:** 10.1371/journal.pone.0273537

**Published:** 2022-08-31

**Authors:** The PLOS ONE Editors

The *PLOS ONE* Editors retract this article [1] because it was identified as one of a series of submissions for which we have concerns about authorship, competing interests, and peer review. We regret that the issues were not addressed prior to the article’s publication.

SIrshad, AM, SIqbal, DI, SK, RMI, MAW, and ZHD did not agree with the retraction. ZH, NR, MN, AAH, and MSE either did not respond directly or could not be reached.
